# Machine Learning Reveals Distinct Immunogenic Signatures of Th1 Imprinting in ART-Treated Individuals with HIV Following Repeated SARS-CoV-2 Vaccination

**DOI:** 10.1101/2025.03.18.643769

**Published:** 2025-03-19

**Authors:** Chapin S. Korosec, Jessica M. Conway, Vitaliy A. Matveev, Mario Ostrowski, Jane M. Heffernan, Mohammad Sajjad Ghaemi

## Abstract

The human immune system is intrinsically variable and remarkably diverse across a population. The immune response to antigens is driven by a complex interplay of time-dependent interdependencies across components of the immune system. After repeated vaccination, the humoral and cellular arms of the immune response display highly heterogeneous dynamics, further complicating the attribution of a phenotypic outcome to specific immune system components. We employ a random forest (RF) approach to classify informative differences in immunogenicity between older people living with HIV (PLWH) on ART and an age-matched control group who received up to five SARS-CoV-2 vaccinations over **104** weeks. RFs identify immunological variables of importance, interpreted as evidence for Th1 imprinting, and suggest novel distinguishing immune features, such as saliva-based antibody screening, as promising diagnostic features towards classifying responses (whereas serum IgG is not). Additionally, we implement supervised and unsupervised *Machine Learning* methods to produce physiologically accurate synthetic datasets that conform to the statistical distribution of the original immunological data, thus enabling further data-driven hypothesis testing and model validation. Our results highlight the effectiveness of RFs in utilizing informative immune feature interdependencies for classification tasks and suggests broad impacts of ML applications for personalized vaccination strategies among high-risk populations.

## Full Text Availability

The license terms selected by the author(s) for this preprint version do not permit archiving in PMC. The full text is available from the preprint server.

